# Molecular architecture of the human citrate synthase–malate dehydrogenase 2 metabolon

**DOI:** 10.1107/S2059798326005802

**Published:** 2026-06-21

**Authors:** Angela J. Kayll, Umanga Rupakheti, Renee St John, Gabriel Rementeria, Joseph J. Provost, Christopher E. Berndsen

**Affiliations:** a Department of Chemistry and Biochemistry James Madison University Harrisonburg VA22807 USA; b Department of Chemistry and Biochemistry University of San Diego San Diego CA92110 USA; Station Biologique de Roscoff, France

**Keywords:** metabolons, SAXS, malate dehydrogenase, citrate synthase

## Abstract

Metabolons are transient biomolecular complexes that enhance the efficiency of metabolic pathways through substrate channeling. These complexes are difficult to study because of their transient nature, thus limiting our understanding of how they are formed and regulated. The citric acid cycle is proposed to contain many such complexes, although few have been characterized structurally. Here, we provide direct structural evidence for the complex of human citrate synthase (hCS) and human mitochondrial malate dehydrogenase 2 (hMDH2), which is part of the larger proposed citric acid cycle metabolon. Our structural model supports previous cross-linking studies and suggests that hMDH2 can interact with each subunit of the hCS dimer, forming up to a hexameric complex. However, this complex appears to be transient, as titration of hMDH2 into hCS in activity assays does not saturate. We further show that the interaction site with hCS is nonspecific, as hCS could also stimulate oxaloacetate formation by cytosolic and plant MDH enzymes. This structural model will provide a basis for understanding the structure and regulation of the broader citric acid cycle metabolon.

## Abstract 2

Human malate dehydrogenase forms a transient complex with citrate synthase.

## 1. Introduction

Cellular metabolism is a web of chemical reactions that must be regulated to produce complex life. Regulation and direction can take many forms, including protein post-translational modification, protein degradation and coupling of enzyme reactions (Sweetlove & Fernie, 2018 ▸; Ciechanover, 2021 ▸; Provost *et al.*, 2024 ▸; Martinez-Vaz *et al.*, 2024 ▸). Malate dehydrogenase (MDH) is a conserved metabolic enzyme found in all branches of life that catalyzes the interconversion of malate and oxaloacetate (OAA) in an NAD(P)(H)-dependent fashion (Woly­niak *et al.*, 2024 ▸; de Lorenzo *et al.*, 2024 ▸). In most animals, MDH is found in the mitochondrion and the cytosol. In other branches of the tree of life, most notably plants, copies of MDH are also found in additional organelles (Baird *et al.*, 2024 ▸; Wolyniak *et al.*, 2024 ▸). In cellular metabolism, the function of MDH is best understood through thermodynamic coupling to citrate synthase (CS), forming a metabolically linked enzyme pair that drives the tricarboxylic acid cycle forward (Omini *et al.*, 2024 ▸). MDH catalyzes the reversible conversion of malate to OAA with the reduction of NAD^+^ to NADH. However, under standard conditions the equilibrium of this reaction strongly favors malate, with a Δ*G*^o^′ of approximately +30 kJ mol^−1^, making OAA formation thermodynamically unfavorable in isolation (Beeckmans & Kanarek, 1981 ▸; Vélot *et al.*, 1997 ▸; Pettersson *et al.*, 2000 ▸). The key to overcoming this barrier lies in the rapid consumption of OAA by CS, which irreversibly condenses OAA with acetyl-CoA to form citrate. This highly exergonic step effectively pulls the MDH reaction forward by depleting OAA, allowing continuous NADH production for oxidative phosphorylation. CS is also a rate-controlling enzyme of the tricarboxylic acid (TCA) cycle cycle, and its demand for OAA ensures that MDH remains functionally integrated into the cycle despite its thermodynamic constraint (Krebs, 1970 ▸; Elcock & McCammon, 1996 ▸; Vélot *et al.*, 1997 ▸; Meyer *et al.*, 2011 ▸; Wu & Minteer, 2019 ▸).

In 1973, Srere and coworkers demonstrated that the immobilization of MDH and CS on a solid matrix enhanced the rate of OAA production, leading to the suggestion that MDH was in close proximity to CS within the mitochondrion, thereby resulting in a locally elevated concentration of OAA (Srere *et al.*, 1973 ▸). Further work with glutaraldehyde cross-linking of MDH and CS resulted in enhanced reaction rates, suggesting the existence of a physical complex between the two proteins (Mattlasson *et al.*, 1974 ▸). Subsequently, Halper and Srere isolated the MDH–CS complex in polyethylene glycol (PEG), exhibiting rate enhancement and specificity of porcine mitochondrial CS for mitochondrial MDH (Halper & Srere, 1977 ▸). In 1985, malate dehydrogenase was isolated as part of a larger complex with several other enzymes from the TCA cycle, including CS (Robinson & Srere, 1985 ▸). The development of models depicting the MDH–CS complex, often involving other enzymes (Elcock & McCammon, 1996 ▸; Vélot *et al.*, 1997 ▸), was subsequently followed by the investigation of fused MDH–CS proteins, which demonstrated enhanced activity in comparison to the free enzymes (Pettersson *et al.*, 2000 ▸; Elcock *et al.*, 1997 ▸). Several studies have shown a direct interaction between MDH and CS through cross-linking and mass spectrometry, leading to proposals for how these proteins interact (Wu & Minteer, 2015 ▸, 2019 ▸; Bulutoglu *et al.*, 2016 ▸; Liu *et al.*, 2017 ▸; Zhang *et al.*, 2017 ▸). Moreover, this complex appears to be evolutionarily conserved, as MDH–CS complexes have been identified in proteins from pigs, mice, humans, yeast and *Bacillus subtilis* (Shatalin *et al.*, 1999 ▸; Meyer *et al.*, 2011 ▸; Bulutoglu *et al.*, 2016 ▸; Jung & Mack, 2018 ▸). The affinity between the two proteins is in the micromolar range, and the association is influenced by metabolites (Omini *et al.*, 2021 ▸). However, observations of a complex revealing the relative orientation of the two enzymes and the stoichiometry of the complex remain absent. This is likely to be attributable to the transient nature of the complex.

Given the support for the MDH–CS complex over the past five decades, one might question the necessity of continuing to investigate the structure of this complex. Currently, it remains uncertain (i) how MDH facilitates the transfer of OAA to CS, potentially via a direct tunnel between the two enzymes, and (ii) how this complex could be subjected to regulation (Pareek *et al.*, 2021 ▸). Both MDH and CS form complexes with numerous other enzymes (Meyer *et al.*, 2011 ▸; Omini *et al.*, 2024 ▸), which may interfere with the formation of citrate or other metabolites; thus, a regulatory mechanism governing complex formation is required, contingent upon cellular status. Prior studies have presented conflicting evidence concerning the role of metabolites, such as ATP, in enhancing complex formation (Omini *et al.*, 2021 ▸). Furthermore, it appears that MDH and CS co-diffuse at elevated rates of malate, a behavior that excess ATP moderates (Wu *et al.*, 2015 ▸). The molecular basis underlying these observations remains unclear.

Considering the unresolved questions about the structure of the MDH–CS complex, we aimed to describe the solution behavior of these two proteins, including their potential interactions and the interaction site. Using small-angle X-ray scattering (SAXS), we characterized a cross-linked, hexameric complex of two human MDH2 molecules and one human CS. We also observed tetrameric forms of the complex, which could accommodate other binding partners. The interaction sites between the two proteins align with conserved surfaces on both proteins and seem to connect the active sites. Interestingly, we also found that we could not saturate CS with MDH in activity assays, suggesting that the hexamer that we observe is a transient association. Additionally, we found that cytosolic MDH enzymes from plants and humans can function with human CS, while an MDH enzyme with a different surface charge density could not. These data support a structural model of the MDH–CS complex in which MDH and CS bind through a charged surface, allowing the transfer of OAA from MDH to CS followed by dissociation of the complex.

## 2. Methods

### 2.1. Purification of hMDH2 and hCS

Human citrate synthase (hCS; UniProt O75390, amino acids 28–466) and *Arabidopsis thaliana* NADP-dependent chloro­plastic MDH (UniProt Q8H1E2, amino acids 58–443), both with a hexahistidine tag, were expressed in *Escherichia coli* cells. hMDH1 splice variant 3 (UniProt P40926-3) was isolated from a human brain cDNA library and cloned into a pET-28 vector. hMDH2 (UniProt P40926-1) was synthesized with codon optimization for expression in *E. coli*. For both human MDH constructs, a TEV protease recognition site and 6×His-tag were included at the C-terminus. The bacterial cell culture was streaked on an LB agar plate with antibiotic overnight in a 37°C incubator. A single colony from a freshly streaked plate was placed into 100 ml 2×YT medium with shaking at 37°C for 24 h. 750 µl of the overnight culture was combined with 0.75 l 2×YT medium and 250 µl 34 mg ml^−1^ kanamycin. The solution was incubated with shaking at 37°C until the culture density reached an OD_600_ of 0.5–0.6. Once this was achieved, the temperature was decreased to 22°C, 0.1 g isopropyl β-d-1-thiogalactopyranoside (final concentration 0.5 m*M*) was added and the culture was incubated overnight with shaking. To harvest the bacterial cells, the medium was centrifuged at 3500*g* for 20 min at 4°C.

The proteins were purified using a multi-step chromatography method on an ÄKTA start FPLC. We resuspended the hMDH2 or hCS bacterial cell pellets in 40 ml of 50 m*M* Tris–HCl, 100 m*M* NaCl, 1 m*M* imidazole, 0.1 m*M* EDTA, along with a Pierce EDTA-free protease-inhibitor tablet and 0.05 g benzamidine. The pellet was warmed in a room-temperature water bath and vortexed until the solution was homogenous. We then sonicated the solution with a microtip at 60% power for three 1 min cycles of 15 s on and 10 s off. We then centrifuged the sample at 17 148*g* for 30 min at 4°C.

Once the pellet had been prepared, we transferred it to an ÄKTA start FPLC. The sample was initially purified with a HisTrap HP 1 ml nickel column. The lysis and wash buffer consisted of 50 m*M* Tris–HCl, 300 m*M* NaCl, 10 m*M* imidazole, 0.1 m*M* EDTA and the elution buffer consisted of 50 m*M* Tris–HCl, 50 m*M* NaCl, 300 m*M* imidazole, 0.1 m*M* EDTA. After loading the soluble lysate, the column was washed with 10 column volumes (CV) of wash buffer. The elution gradient was a linear gradient to 5% elution buffer in 10 CV, then to 40% elution buffer in 20 CV and then to 100% elution buffer in 10 CV. All proteins eluted between 50 and 150 m*M* imidazole. Afterwards, we observed the UV absorbance of the fractions and confirmed the presence of protein in the sample using SDS–PAGE. Samples with protein of the appropriate molecular weight were centrifuged in 15 ml concentrator tubes with a molecular-weight cutoff of 3.5 kDa at 4°C until the sample volume was approximately 1.5 ml.

Following concentration, we further purified the proteins with a Sephacryl 16/60 S100 size-exclusion column for hMDH2 and a Sephacryl 16/60 S200 size-exclusion column for hCS in 50 m*M* Tris–HCl, 100 m*M* NaCl, 0.1 m*M* EDTA. Afterwards, we confirmed the presence of hMDH2 or hCS in the sample using a BioTek Eon spectrophotometer. Samples with protein present, based on a band at the appropriate molecular weight, were centrifuged in a 15 ml concentrator at 4°C until the sample volume was approximately 1.0 ml. The protein concentration was determined using extinction coefficients of 8940 *M*^−1^ cm^−1^ for hMDH2 and 76 320 *M*^−1^ cm^−1^ for hCS on a Take-3 plate on a BioTek Eon spectrophotometer.

### 2.2. Cross-linking of hMDH2 and hCS

200 µl 20 m*M* MES pH 6.4, 900 µl 1.3 mg ml^−1^ hCS, 300 µl 8.1 mg ml^−1^ hMDH2 and 1100 µl nanopure H_2_O were combined and incubated at room temperature for 15 min. 300 µl 0.5% glutaraldehyde stock was combined with the protein solution and rapidly vortexed. The solution was incubated at room temperature for 30 min. We added 300 µl 1 *M* Tris pH 8 to quench excess glutaraldehyde and then incubated the sample at room temperature for approximately 5 min. We then purified the sample with an ÄKTA start FPLC and a Sephacryl 16/60 S200 size-exclusion column in 50 m*M* Tris–HCl pH 7.5, 100 m*M* NaCl, 0.1 m*M* EDTA. Fractions containing cross-linked protein were identified by SDS–PAGE and concentrated in 15 ml concentrator tubes with a molecular-weight cutoff of 3.5 kDa at 4°C.

### 2.3. Analytical SEC

We injected 50 µl cross-linked hMDH2–hCS onto a Wyatt 5 µm 300 Å 4.6 mm internal diameter SEC analytical column equilibrated in 20 m*M* sodium phosphate, 50 m*M* NaCl buffer pH 8.0. The flow rate was 0.4 ml min^−1^. The molecular weights shown in the figure were calculated based on the elution times of proteins in the Cytiva high- and low-molecular-weight kits. Standards were also used to generate a standard curve to calculate the molecular weight of the cross-linked protein complex.

### 2.4. Small-angle X-ray scattering

For hCS, we used SEC-SAXS and HT-SAXS to describe the structure, while we used SEC-SAXS for the hMDH2–hCS cross-linked sample. hCS was diluted to a concentration in the range 0.07–2.0 mg ml^−1^ in size-exclusion buffer (50 m*M* Tris–HCl, 100 m*M* NaCl, 0.1 m*M* EDTA pH 7.5) and then placed into a 96-well plate. The samples were frozen at −80°C before shipment. Data were collected on the SIBYLS beamline 12.3.1 at the Advanced Light Source, Lawrence Berkeley National Laboratory (Classen *et al.*, 2013 ▸; Rosenberg *et al.*, 2022 ▸). Before sample collection, the sample plate was spun at 2500*g* for 10 min. Samples were held at 10°C during collection. The exposure was 15 s, with frames collected every 0.3 s for 40 frames per sample. The incident light wavelength was 1.127 Å at a sample-to-detector distance of 2.1 m. This setup results in scattering vectors, *q*, ranging from 0.013 to 0.5 Å^−1^, where the scattering vector is defined as *q* = 4πsinθ/λ and 2θ is the measured scattering angle.

For SEC-SAXS, samples were separated on a Shodex KW-803 column at a flow rate of 0.5 ml min^−1^ at 10°C and eluate was measured in-line with UV–Vis absorbance at 280 nm, multi-angle X-ray scattering (MALS) and SAXS. The column buffer was 50 m*M* sodium phosphate pH 7, 100 m*M* NaCl, 2 m*M* DTT, 2% glycerol. The incident light wavelength was 1.03 Å at a sample-to-detector distance of 2.1 m.

To process SEC-SAXS data, *CHROMIXS* was used to subtract buffer from the peaks and produce the initial data file (Panjkovich & Svergun, 2018 ▸; Manalastas-Cantos *et al.*, 2021 ▸; Franke *et al.*, 2025 ▸). We then used *RAW* to analyze the data and generate statistics for the processed SEC-SAXS and HT-SAXS data and *FoXS* to fit structural models to the SAXS data (Schneidman-Duhovny *et al.*, 2013 ▸; Hopkins, 2024 ▸). The conformation of the hCS model was explored using *NRGTEN* and the models were fitted to the data using *FoXS* (Mailhot & Najmanovich, 2021 ▸). The SAXS data and models have been deposited in the SASBDB under codes SASDX87 and SASDX97 for hCS and SASDXA7 for the hMDH2–hCS complex.

### 2.5. LightDock

A dimer of hCS and hMDH2 dimers was constructed in *LightDock* using cross-linking information from previous studies (Jiménez-García *et al.*, 2018 ▸; Roel-Touris *et al.*, 2020 ▸). The dimer of dimers was converted to a trimer of dimers via a twofold rotation around the CS subunit. The resulting hexamers were fitted to the SAXS data using *FoXS*.

### 2.6. Coupling of MDH and CS activity

Assays coupling hCS and hMDH2 activity were performed in 50 m*M* sodium phosphate pH 8.0 with 100 µ*M* malate, 100 µ*M* NAD^+^, 25 µ*M* acetyl-CoA. Assays were run with 0.3 µ*M* hCS and 0.4 µ*M* hMDH2 or 0.3 µ*M* of the cross-linked complex or 0.4 µ*M* of hMDH2 alone. Assays were performed in duplicate simultaneously. Assays were performed in a 96-well plate and data were collected using a BioTek Eon spectrophotometer at 340 nm. Data were collected every 4–6 s for 10 min. MDH activity alone was confirmed in assays with 100 µ*M* OAA and NADH instead of malate, NAD^+^ and acetyl-CoA. MDH inactivity was confirmed in assays with 100 µ*M* malate and NAD^+^ with no citrate synthase or acetyl-CoA, following the same data-collection parameters.

### 2.7. Titration of hMDH2–hCS activity

Reactions contained 50 m*M* sodium phosphate buffer pH 8, 10 m*M* acetyl-CoA, 5 m*M* malate, 3.9 m*M* NAD, 2.5 µ*M* hCS. The hMDH2 concentration was varied to achieve hMDH2:hCS molar ratios of 0:1, 0.25:1, 0.5:1, 1:1, 1.5:1, 2:1 and 3:1. The hCS concentration was held constant. Final reactions were prepared by combining 40 µl buffer, 1 µl malate, 4 µl acetyl-CoA, 3 µl hCS and corresponding volumes of hMDH2 in a final volume of 250 µl. Assays were performed using a BMG Labtech CLARIOstarPlus device with Smart Control app at 25°C. To initiate reactions, 6 µl NAD^+^ was added to each well, followed immediately by 200 µl reaction mixture. Control reactions without the enzyme were included to account for background absorbance changes. Activity was measured at 340 nm for 150 cycles with 15 flashes per cycle and a cycle time of 5 s. Initial rates were calculated from the linear portion of the data and the data were analyzed and visualized in *R*.

## 3. Results

### 3.1. Structure of hCS

**Figure 1:**
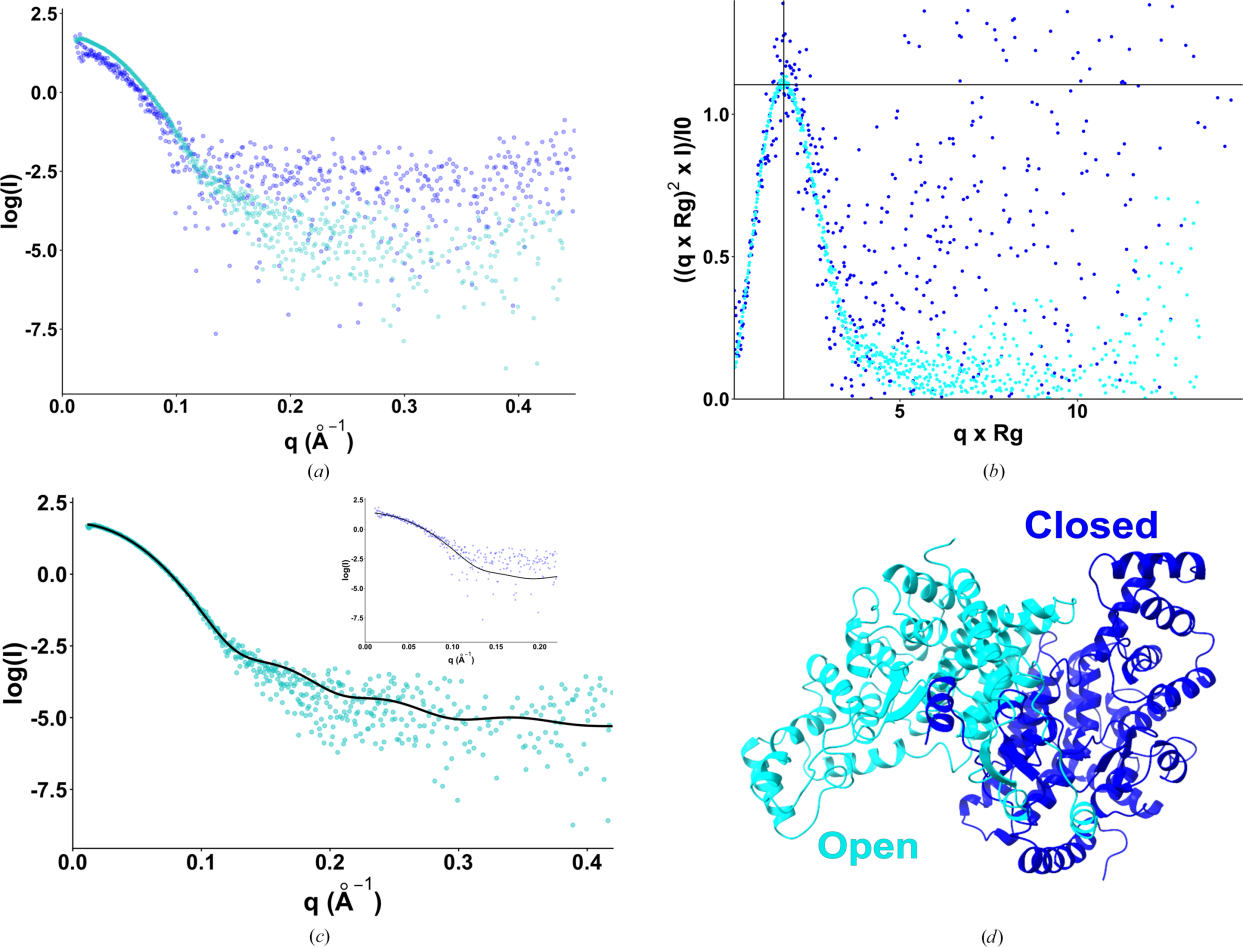
Small-angle X-ray scattering of hCS. (*a*) The log(*I*) versus *q* plot for two data sets of hCS. (*b*) Kratky plot of the data. (*c*) Fitting of the modeled structure in (*d*) to SAXS data sets. The χ^2^ value was 0.7 (main plot) and 1.6 (inset plot). The cyan subunit is in the open conformation, while the blue subunit is in the closed conformation. The starting model prior to refinement by normal-mode analysis was PDB entry 5uzr (Schlachter *et al.*, 2019 ▸).

While the crystal structures of hMDH2 and hCS and their ability to interact are known (Bulutoglu *et al.*, 2016 ▸; Schlachter *et al.*, 2019 ▸; Berndsen *et al.*, 2025 ▸), how these proteins associate and combine to form a metabolon is less clear. Before we could describe the complex, we first needed to conform the structure of hCS. Citrate synthase has not been characterized in solution via SAXS before, while the solution structure of hMDH2 has recently been described (Berndsen *et al.*, 2025 ▸). Thus, we needed to ensure that the X-ray crystal structures matched the solution behavior of these proteins to develop an accurate model of the complex. We compared our SAXS data on hCS with known crystal structures, finding that hCS appeared to form a dimer in solution but the conformation of CS in crystal structures did not fully match our solution data (Schlachter *et al.*, 2019 ▸; Supplementary Fig. S1). Because hCS is likely to undergo conformational changes (Bayer *et al.*, 1981 ▸; Schlachter *et al.*, 2019 ▸), we sampled additional conformations of CS using normal-mode analysis and found a conformation that fitted both data sets with a χ^2^ of 0.7 and 1.6 (Fig. 1 ▸; Mailhot & Najmanovich, 2021 ▸). In this conformation, one subunit of CS is in the open conformation with the active site accessible, while the other is in the closed conformation, which has previously been associated with a substrate-bound conformation (Schlachter *et al.*, 2019 ▸; Fig. 1 ▸).

### 3.2. Formation of the complex between hMDH2 and hCS

**Figure 2:**
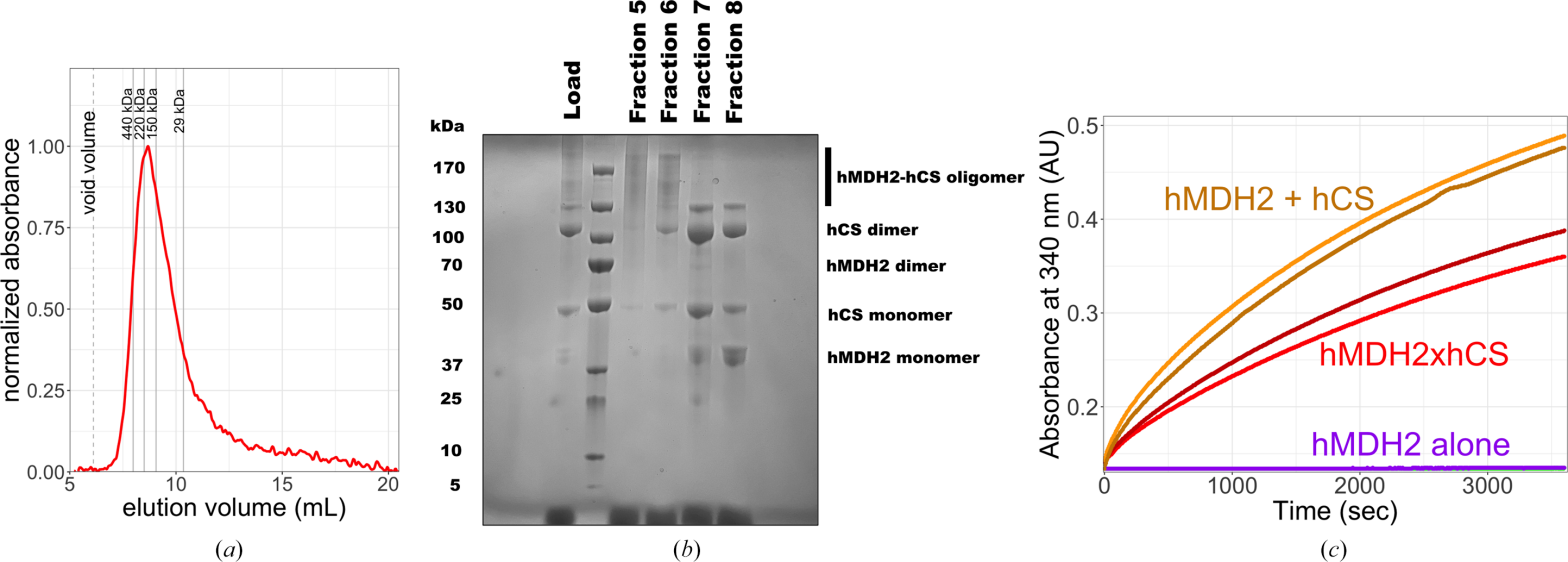
Purification of a cross-linked hMDH2–hCS complex. (*a*) Size-exclusion chromatography of the cross-linked complex relative to the elution volumes of standard proteins. (*b*) Gel of the main fractions across the peak in (*a*). (*c*) NAD^+^-reduction activity of the cross-linked complex (hMDH2xhCS; red) relative to a 1:1 mixture of hMDH2 and hCS (orange) and hMDH2 alone (purple).

Having more confidence in the solution structures of hCS and hMDH2, we next worked to characterize the structure of the complex via SAXS. We initially tried to mix the proteins and purify the complex by size-exclusion chromatography. However, we failed to observe a prevalent and distinct complex of the two proteins (data not shown). Thus, we turned to glutaraldehyde cross-linking of the proteins to stabilize a potentially transient complex. We then purified the complex via preparative SEC followed by molecular-weight determination by analytical SEC (Fig. 2 ▸*a*). We observed a single peak and found that the peak elution volume was between the 150 and 220 kDa standard protein elution times, supporting the findings from the SDS–PAGE experiments. The calculated molecular weight was 187 kDa, suggesting a mixture of hexameric and tetrameric complexes. We separated fractions on SDS–PAGE to see the resulting species. As shown in Fig. 2 ▸(*b*), we observed a smear of bands consistent with a larger molecular-weight complex of hCS and hMDH2. In fractions 5 and 6, this smear spanned from the 130 kDa marker to species larger than 170 kDa (Fig. 2 ▸*b*). Others have previously observed these higher molecular-weight species using longer cross-linking reagents, suggesting that this complex is not an artifact of our system (Bulutoglu *et al.*, 2016 ▸). These data suggest that hMDH2 and hCS can form a tetrameric or hexameric complex. Moreover, these data support a structural model in which hCS has two possible interaction sites with hMDH2.

### 3.3. Cross-linked hMDH2–hCS can form NADH and oxaloacetate

To confirm that the semipurified, larger molecular-weight protein complex observed in SDS–PAGE and analytical SEC was the hMDH2–hCS protein complex, and that cross-linking did not affect its function, we assayed the activity of the cross-linked fraction compared with a non-cross-linked sample. The SEC-purified fractions in Fig. 2 ▸(*b*) appear to lack significant amounts of free hMDH2; thus, we felt confident that activity would be due to species in complex and not free enzyme. In NAD^+^-reduction assays, we compared the activity from solutions of hMDH2 and hCS with no glutaraldehyde cross-linking, cross-linked hMDH2 and hCS, and hMDH2 alone (Fig. 2 ▸*c*). hMDH2 alone showed relatively little activity, suggesting that it could not measurably reduce NAD^+^ in these assays. The cross-linked sample had activity levels within twofold of hMDH2 and hCS without cross-linking (Fig. 2 ▸*c*). These data indicate that the cross-linked complex can generate NADH and oxaloacetate, the latter of which was likely used by hCS to produce citrate, although this was not directly measured. Previous studies on complexes linked by longer cross-linkers have shown similar activity (Shatalin *et al.*, 1999 ▸; Bulutoglu *et al.*, 2016 ▸). Therefore, any structural information obtained from this sample will probably reflect the catalytically active configuration of the complex.

### 3.4. Structure of the complex between hMDH2 and hCS

**Figure 3:**
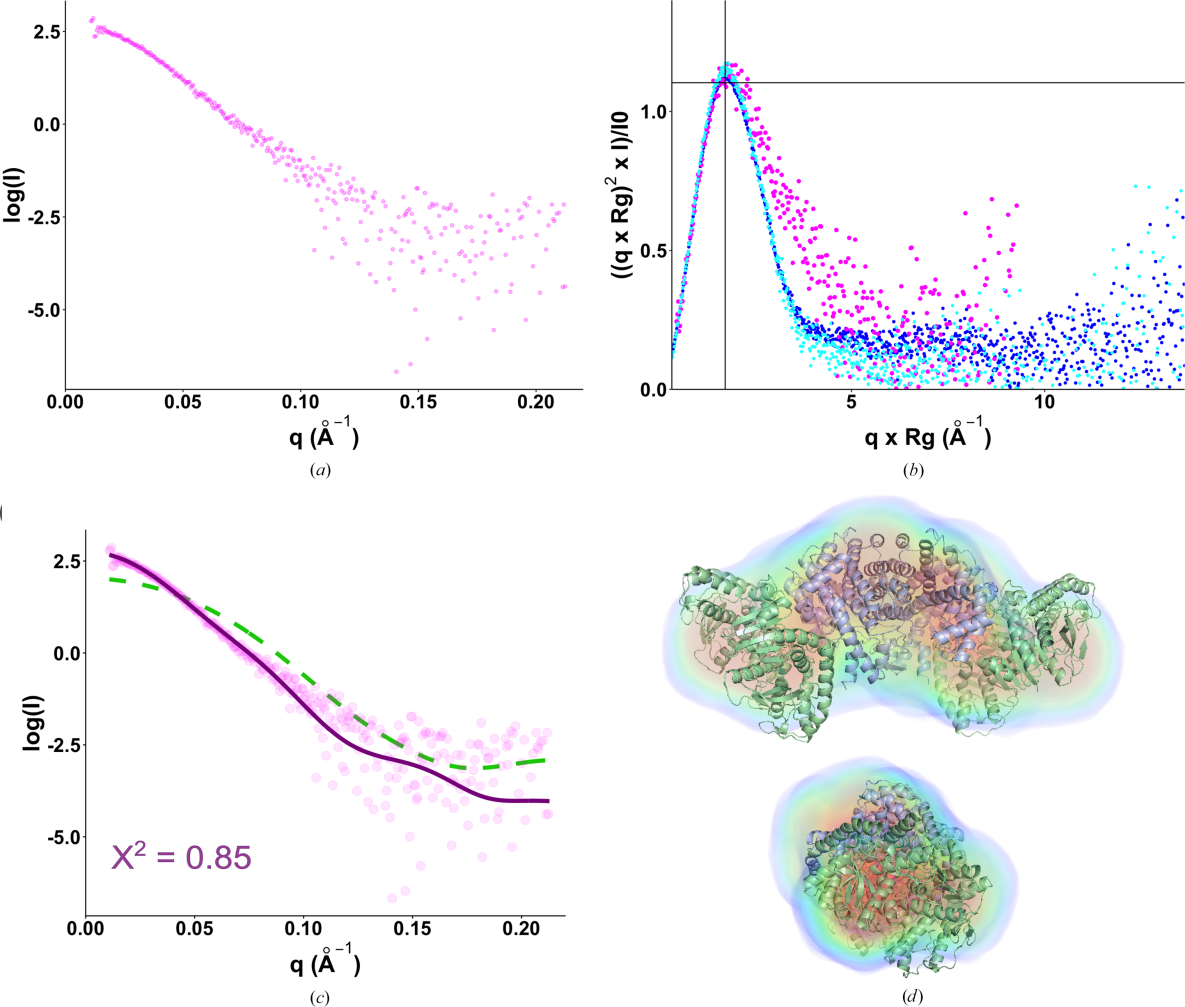
Small-angle X-ray data for the hMDH2–hCS complex. (*a*) The log(*I*) versus *q* plot of the trapped hMDH2–hCS complex. (*b*) Comparison of hCS data in Kratky form from Fig. 2 ▸ (blue/cyan) to the complex showing the differences in shape. (*c*) *FoXS* fitting of the *AlphaFold*3 model of the MDH–CS hexamer (green, χ^2^ = 2.3) compared with the final best-fitting model derived from *LightDock* fitting (purple, χ^2^ = 0.85). (*d*) Electron density of the best-fitting MDH–CS hexamer derived from *DENSS* aligned with the best-fitting hexamer model from (*c*). The mean real-space correlation was 0.85 ± 0.04 and the Fourier shell correlation resolution was 55.6 ± 7.3 Å.

We next collected HT-SAXS and SEC-SAXS data on the cross-linked MDH–CS. The HT-SAXS data showed evidence of higher molecular-weight species, which we observed in the void region in the SEC-SAXS data. Thus, we used the SEC-SAXS data to model the configuration of the complex. The Kratky plots for the hMDH2–hCS complex, hMDH2 and hCS indicated compact, globular shapes (Fig. 3 ▸ and Table 1 ▸). When comparing SAXS data from hMDH2 and hCS with the hMDH2–hCS complex data, the higher values for the radius of gyration (*R*_g_; hMDH2–hCS, 43.8 Å; hCS, 30.7 Å; hMDH2, 28.1 Å), molecular weight (MW; hMDH2–hCS, 220.7 kDa; hCS, 96.1; hMDH2, 68.8 kDa) and maximum length (*D*_max_; hMDH2-hCS, 150 Å; hCS, 99 Å; hMDH2, 91 Å) observed for the complex suggest that the cross-linked samples are in an oligomeric state. If the sample contained a significant mixture of the free hMDH2 and hCS, we would expect these values to be between the values for isolated proteins. The data are more consistent with a hexameric species of hMDH2–hCS with a predicted molecular weight of 237 kDa.

### 3.5. Modeling the complex between hMDH2 and hCS

Initial attempts to use *AlphaFold*3 to generate a tetrameric or hexameric complex resulted in complexes that fitted poorly to the SAXS data (Abramson *et al.*, 2024 ▸; Fig. 3 ▸*c*). We then used *LightDock* to create a tetrameric complex of hMDH2 and hCS with the restraint that Arg63 and Arg65 of hCS had to be adjacent to the interaction site to facilitate the transfer of OAA from hMDH2 to the active site of hCS (Jiménez-García *et al.*, 2018 ▸). When we fitted the tetramer models to the SAXS data in *FoXS*, we observed fits with χ^2^ values greater than 1.4 and which required extreme hydration layer values (Supplementary Fig. S2). We then created hexameric complexes from the tetramers by mirroring the MDH dimer across the hCS homodimer and fitting the models to the SAXS data. The fit of the hexameric model to the data had a χ^2^ of 0.85. We further fitted the data using *OLIGOMER* and *Multi-FoXS* to determine the ratio of dimeric, tetrameric and hexameric species in our data (Konarev *et al.*, 2003 ▸; Schneidman-Duhovny *et al.*, 2016 ▸). From both methods, there was minimal enhancement of the fit quality through the addition of tetrameric or dimeric species to the data (χ^2^ of 0.85 versus 0.84; Supplementary Fig. S3). Thus, we were confident that the hexameric complex was the primary species in our sample. This configuration of hCS and hMDH2 in the model puts the OAA-binding sites in hMDH2 and hCS from crystal structures 34 Å apart. The active site of hMDH2 aligns with the ‘basic pocket’ surrounding the hCS active site, which is necessary to guide OAA to the active site of hCS (Elcock & McCammon, 1996 ▸; Elcock *et al.*, 1997 ▸; Wu & Minteer, 2015 ▸; Bulutoglu *et al.*, 2016 ▸).

**Figure 4:**
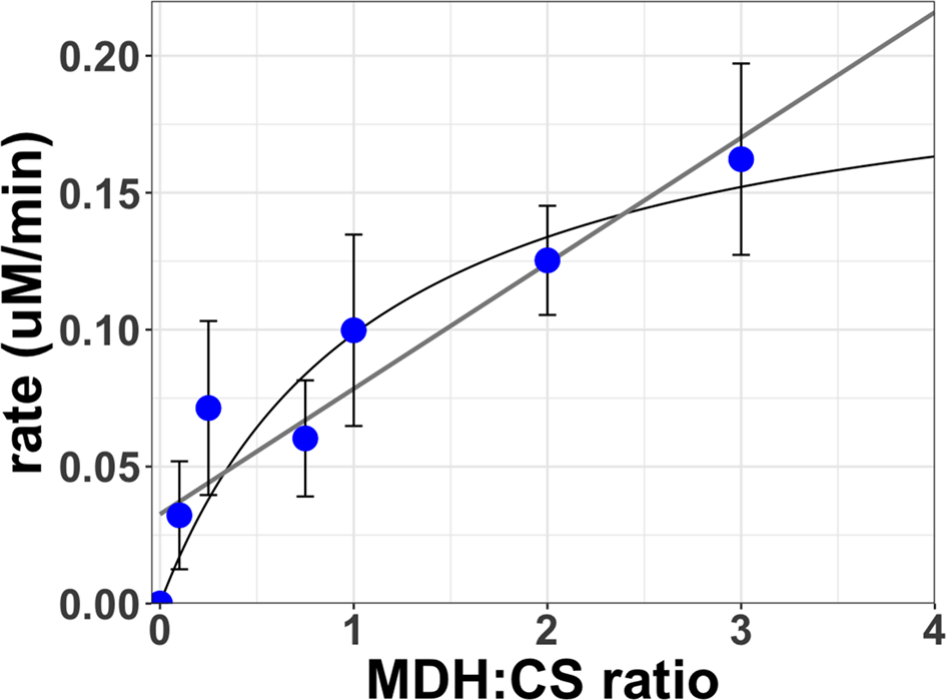
Titration of hMDH2 in activity assays. Blue dots are average values from at least three independent assays, with error bars showing the standard deviation. The hyperbolic and linear fitting lines are shown, and the *R*^2^ values were 0.86 for the hyperbolic fit and 0.66 for the linear fit.

To support the stoichiometry suggested by the structural data, we titrated hMDH2 into hCS and measured the production of NADH in enzyme assays. Given that hMDH2 cannot produce significant amounts of NADH in the absence of hCS (Fig. 2 ▸*c*), this assay setup is sensitive to formation of the hMDH2–hCS complex. In fitting the data to a hyperbolic model, the data show a linear rise in activity until the hMDH2:hCS ratio reaches 1:1, followed by a gradual decrease in the steepness of the rise (Fig. 4 ▸). However, we noticed that a linear fit to the data was also visually reasonable. One caveat is that the presence of hCS can stimulate hMDH2 activity, as shown in Fig. 2 ▸(*c*), without cross-linking; thus, we cannot say for certain that we are saturating hCS with hMDH2, especially since the affinity of hCS for OAA is reportedly higher than the affinity of hMDH2 for OAA (Mukherjee *et al.*, 1980 ▸). With the SAXS and SEC data, we suggest these data support a potential >1:1 ratio of hMDH2 dimer to hCS dimer.

### 3.6. Conservation around the interaction surface

**Figure 5:**
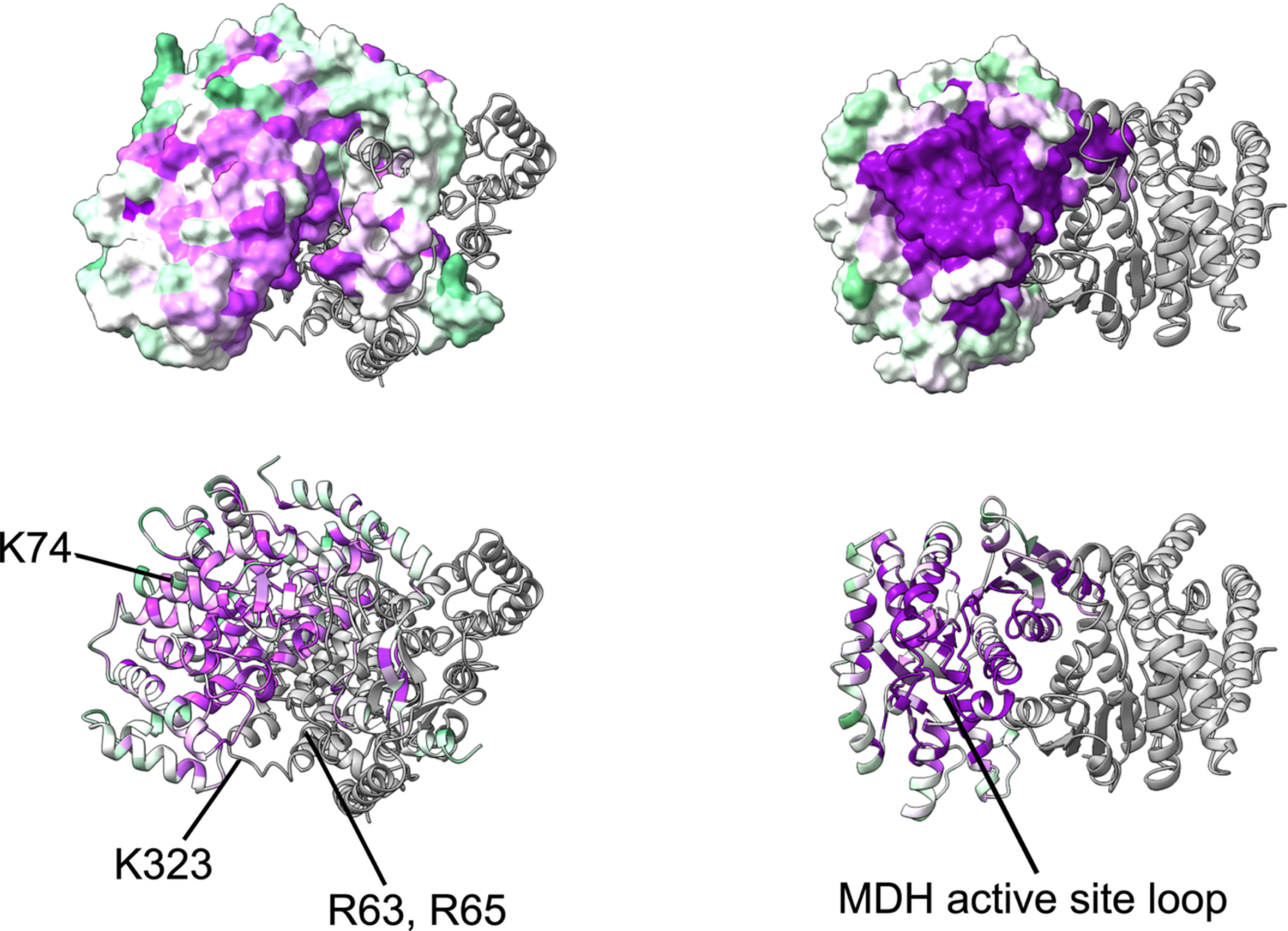
Conservation of the complex interaction surface for hCS (left) and hMDH2 (right). Identical amino acids in *ConSurf* analysis are indicated with purple coloring. Green shades indicate regions with 60–90% conservation, while white areas have less than 50% sequence conservation. Known interaction-site side chains within hCS from previous work are indicated (Bulutoglu *et al.*, 2016 ▸).

We then used *ConSurf* to identify conserved surfaces of hMDH2 and hCS (Ashkenazy *et al.*, 2016 ▸). To improve the results, we curated the sequences for each enzyme to only include known mitochondrial MDH and CS homologs and used the same species in each dataset (Supplementary Figs. S4 and S5). The interaction site within hCS shows greater conservation than other surfaces on CS (Fig. 5 ▸). Moreover, this conserved patch includes the known interaction site and previously identified cross-linking sites (Bulutoglu *et al.*, 2016 ▸). In hMDH2, the interaction is conserved and includes the active site of hMDH2. Thus, the atomic-level model of hMDH2–hCS is consistent with the SAXS data and is plausible based on biochemical and sequence conservation data.

### 3.7. The hCS complex binding site is not sequence-specific

**Figure 6:**
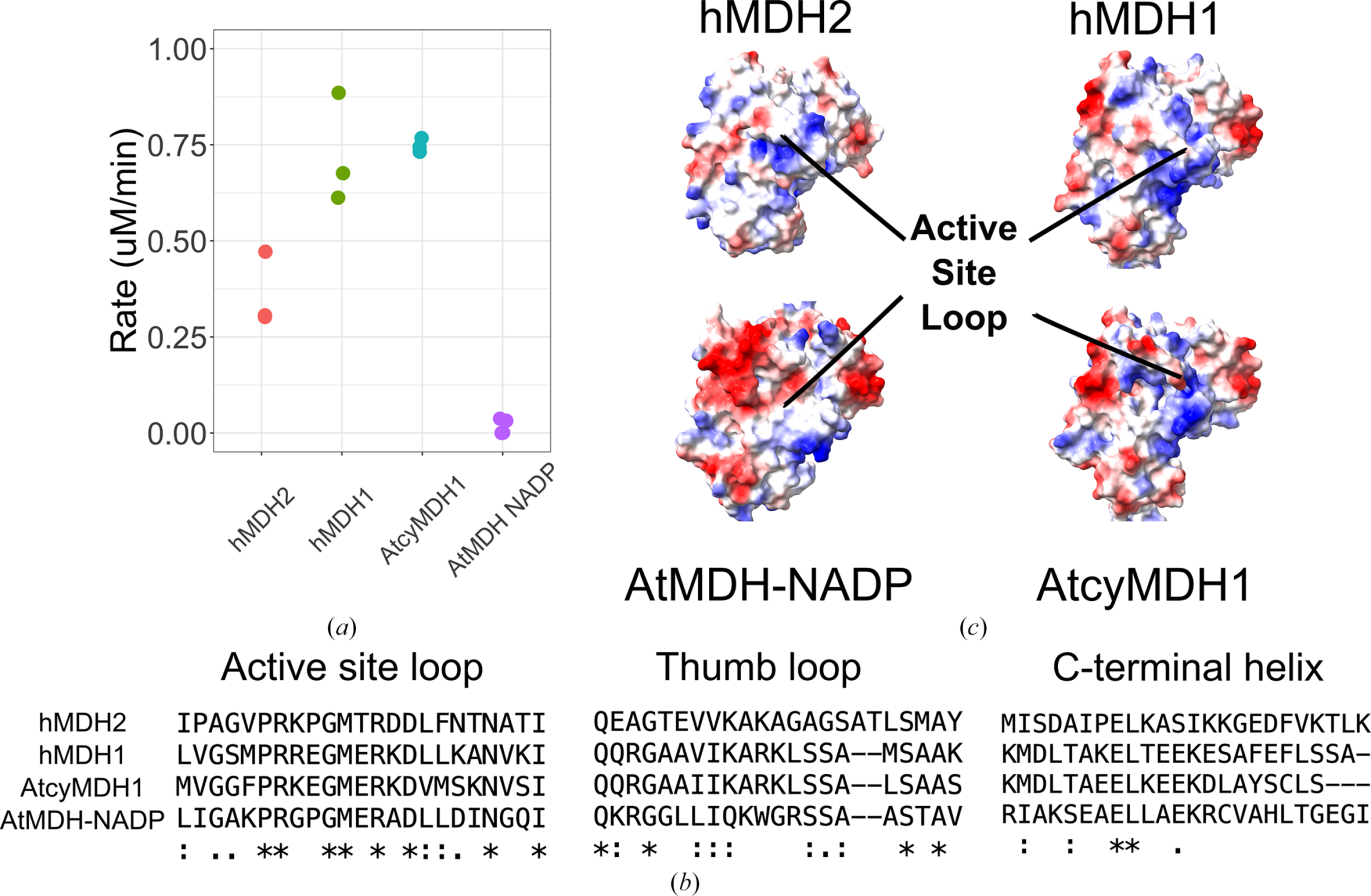
Interaction with hCS is based on surface charge. (*a*) Assays of several MDH enzymes with hCS. Rate values from three separate assays are shown. (*b*) Conservation of the sequences of the active-site loops and C-terminal helix in the hCS interaction site from *Clustal Omega*. (*c*) Surface charge of the hCS site in the MDH enzymes used in (*a*). Red indicates negatively charged amino-acid side chains, while blue colors indicate positively charged side chains.

Given our findings that the hMDH2–hCS complex appears to be transient, we wanted to test the specificity of the interaction. First, we measured the OAA-forming activity of hMDH1 in the presence of hCS, finding that it was stimulated by hCS (Fig. 6 ▸). We then expanded the specificity experiment to include nonmitochondrial plant MDHs: the cytosolic MDH1 (AtMDH1) and the chloroplastic, NADP-dependent MDH, both from *Arabidopsis thaliana*. We found that hCS also stimulated AtMDH1 activity, but that the NADP-dependent MDH was not stimulated even in the presence of NADP^+^. A homolog of this protein from *Chlamydomonas* is reported to have a *K*_m_ value of ∼36 µ*M* for OAA (Lemaire *et al.*, 2005 ▸), greater than that reported for hCS (Mukherjee *et al.*, 1980 ▸). The *K*_m_ values for OAA of mammalian MDH1 are reportedly in the 20–60 µ*M* range (Telegdi *et al.*, 1973 ▸; Bernstein *et al.*, 1978 ▸; Crow *et al.*, 1983 ▸; de Lorenzo *et al.*, 2024 ▸) and that of the cytosolic MDH1 from *Arabidopsis* is 238 µ*M* (Huang *et al.*, 2018 ▸). The data and previous work together suggest that not all MDH enzymes are compatible with hCS, which we attribute to the inability to form a complex. Sequence comparison of all four enzymes showed limited sequence conservation of the interaction surfaces near the active site and the C-terminal regions implicated in binding to hCS. Prediction of the electrostatic surfaces of each of the enzymes showed a larger patch of negative charge and less positive charge in the NADP-dependent MDH that was not seen in the hMDH2 or the cytosolic MDH proteins (Fig. 6 ▸*c*). These data support previous findings that the interaction between MDH and hCS is partly defined by charge complementarity at the binding surface (Elcock & McCammon, 1996 ▸; Elcock *et al.*, 1997 ▸; Shatalin *et al.*, 1999 ▸; Wu & Minteer, 2015 ▸; Bulutoglu *et al.*, 2016 ▸).

## 4. Discussion

Interactions between adjacent enzymes in metabolic pathways are known to enhance the flow of metabolites and, in the case of hMDH2, help to overcome unfavorable thermodynamic barriers (Sweetlove & Fernie, 2018 ▸; Omini *et al.*, 2024 ▸). The structures and configurations of these complexes have been elusive, partly because of the inability to purify stable species. Previous work on hMDH2 and hCS has focused on the conditions that promote contact or identifying interaction sites (Halper & Srere, 1977 ▸; Bulutoglu *et al.*, 2016 ▸; Omini *et al.*, 2021 ▸). However, how the two dimers assembled remained unclear. Thus, our work clarifies these previous studies to produce a structural model of the interaction consistent with the cross-linking studies and adds to our understanding of the complex. Here, we used glutaraldehyde cross-linking to stabilize a hexameric complex of hCS and hMDH2. This complex can form OAA *in vitro*, and similar complexes with masses of 170 kDa or more have been seen by others (Bulutoglu *et al.*, 2016 ▸). We further described the solution structure of hCS, finding that the crystal structures do not fit the data well due to asymmetry in the structure which is not fully seen in the crystal structures (Schlachter *et al.*, 2019 ▸). These data suggest a structural reaction mechanism for hCS that may involve cycling between conformations; however, this is not fully clear from the SAXS data presented. Modeling of the complex to SAXS data, guided by previous cross-linking studies, revealed that a hexameric model best fits the data (Figs. 4 ▸ and 5 ▸; Wu & Minteer, 2015 ▸; Bulutoglu *et al.*, 2016 ▸). We then showed that the interaction surface is not species- or family-specific, as hCS could promote OAA formation for cytosolic MDH enzymes from both humans and *Arabidopsis*. Our predicted interaction surface supports previous work showing that ionic strength was a strong regulator of complex activity, suggesting that the interaction site is charged and polar (Shatalin *et al.*, 1999 ▸).

From the structural model, the active sites of MDH and CS appear to ‘pair off’, with a single MDH active site within the dimer interacting with a single active site within the CS dimer. This configuration maximizes the chances that OAA dissociating from MDH is proximal to the entry path of the CS active site (Bulutoglu *et al.*, 2016 ▸; Schlachter *et al.*, 2019 ▸). Furthermore, this configuration is consistent with the reported reciprocal or half-the-sites behavior of the MDH dimer reported in the literature (Harada & Wolfe, 1968 ▸; Berndsen & Bell, 2024 ▸). In the reciprocal mechanism, only one active site in the MDH dimer is active at a time. In addition, CS is known to undergo significant structural rearrangements during the catalytic cycle near the interface with MDH (Bayer *et al.*, 1981 ▸; Schlachter *et al.*, 2019 ▸). Our SAXS data on hCS appear to further support this, as a model that was in a half-open/half-closed state appeared to fit the data better than the crystal structures. The structural rearrangements in both proteins are not blocked by interaction, as we and others show that cross-linked MDH–CS is an active complex (Bulutoglu *et al.*, 2016 ▸; Shatalin *et al.*, 1999 ▸). Thus, our proposed arrangement of hMDH2 and hCS is consistent with the catalytic cycle of both proteins.

The complex that we describe differs from the configurations proposed based on mass spectrometry and bio­informatics (Vélot *et al.*, 1997 ▸; Bulutoglu *et al.*, 2016 ▸). Even *AlphaFold*3 predicts a distinct configuration from that which best fits our SAXS data. However, our predicted model does satisfy the contacts suggested by MS and does orient the active sites more closely than some of these models (Bulutoglu *et al.*, 2016 ▸). Further, we provide higher resolution information by observing the predominant species in a solution of purified cross-linked protein via SAXS. Thus, we consider our model a refinement of these earlier models based on new data, rather than presenting an alternative complex.

While we observed a hexameric species in SAXS, we also observed tetrameric species in other experiments, suggesting that both configurations are possible and functional. The trimer-of-dimers configuration is likely due to the excess hMDH2 that we used in the cross-linking reactions. Titration experiments of hMDH2 into hCS, monitoring NADH production, showed that hCS did not fully saturate under our experimental conditions, suggesting that complex formation is more transient. The historic difficulty in purifying this complex without cross-linking, along with the low interaction-site specificity, support a model in which MDH enzymes are rapidly transferring OAA to CS. Our findings further support recent work by Omini and coworkers and suggest a rapidly exchanging complex in yeast (Omini *et al.*, 2025 ▸). This rapidly exchanging system would allow faster regulation of the system and simplifies the interaction site for a wide range of partners. Thus, our hexameric model is likely to represent a snapshot of a highly dynamic system and the maximally loaded state.

Since multiple interaction interfaces between malate dehydrogenase and citrate synthase have been observed, the factors that contribute to the stabilization or selective formation of specific MDH–CS complexes *in vivo* remain open questions. Additionally, how post-translational modifications might influence the assembly, orientation or regulation of these metabolons is also under investigation. hCS is known to be methylated at Lys366 (Lys395 before processing), decreasing activity, and this modification site is adjacent to the interface with hMDH2 (Małecki *et al.*, 2017 ▸; Rhein *et al.*, 2017 ▸). Several large-scale studies have identified acetylation, phosphorylation and succinylation of amino acids near the interaction site in both hCS and hMDH2, which would neutralize or invert the charge at the interaction surface (Zhao *et al.*, 2010 ▸; Prus *et al.*, 2024 ▸). In light of data suggesting charge complementarity as key for the interaction of hMDH2 with hCS, these modifications would likely disrupt complex formation and activity. Further work is needed to confirm whether the other interaction site of hCS partners overlaps with that described here; however, MDH and aspartate aminotransferase are antagonistic in activity assays, suggesting a common surface (Beeckmans & Kanarek, 1981 ▸; Fahien *et al.*, 1988 ▸; Elcock *et al.*, 1997 ▸).

## Supplementary Material

SASBDB reference: human citrate synthase, SASDX87

SASBDB reference: SASDX97

SASBDB reference: complex with human mitochondrial malate dehydrogenase 2, SASDXA7

Supplementary Figures. DOI: 10.1107/S2059798326005802/jc5067sup1.pdf

## Figures and Tables

**Table 1:** SAXS statistics

	hCS		
	High concentration	Low concentration	hMDH2–hCS
Wavelength (Å)	1.127	1.127	1.127
*q*-range (Å^−1^)	0.0098–0.4707	0.0099–0.4707	0.0124–0.4707
Concentration (mg ml^−1^)	2	4	1
Temperature (K)	283	283	283
*I*(0) (AU) [from *p*(*r*)]	6.0 ± 0.1	4.1 ± 0.1	15.2 ± 0.2
*R*_g_ (Å) [from *p*(*r*)]	30.8 ± 0.1	30.3 ± 0.7	45.6 ± 0.5
*I*(0) (AU) (from Guinier)	5.9 ± 0.1	4.2 ± 0.3	14.9 ± 0.3
*R*_g_ (Å) (from Guinier)	30.6 ± 0.6	31.2 ± 4.2	43.8 ± 0.9
*D*_max_ (Å)	106	96	153
Porod volume estimate (*V*_p_) (Å^3^)	131000	113000	266000
Molecular mass, Bayes (kDa)	105 (90.7% CI 99–111)	86 (92% CI 75–87)	208 (97% CI 195–264)
Molecular mass *M*_r_ (from Porod volume) (kDa)	108	94	221
Calculated monomeric *M*_r_ from sequence (kDa)	49	49	82
SASBDB code	SASDX97	SASDX87	SASDXA7
χ^2^ of *FoXS* fit model	1.6	0.7	0.85
Primary data reduction	*RAW*	*RAW*	*RAW*
Data processing	*RAW*	*RAW*	*RAW*
Computation of model intensities	*FoXS*	*FoXS*	*FoXS*
Three-dimensional graphic representations	*ChimeraX*	*ChimeraX*	*ChimeraX*
