# Supplementary material for: Molecular architecture of the human citrate synthase–malate dehydrogenase 2 metabolon
Source: Acta Crystallogr D Struct Biol. 2026 Jun 21;82(Pt 7):740–50. doi: 10.1107/S2059798326005802 (PMC13317680; doi:10.1107/S2059798326005802)
Supplement: Supplementary file 1 [file d-82-00740-sup1.pdf]

STRUCTURAL BIOLOGY

Volume 82 (2026)

Supporting information for article:

Molecular architecture of the human citrate synthase–malate dehydrogenase 2 metabolon

Angela J. Kayll, Umanga Rupakheti, Renee St John, Gabriel Rementeria, Joseph J. Provost and Christopher E. Berndsen

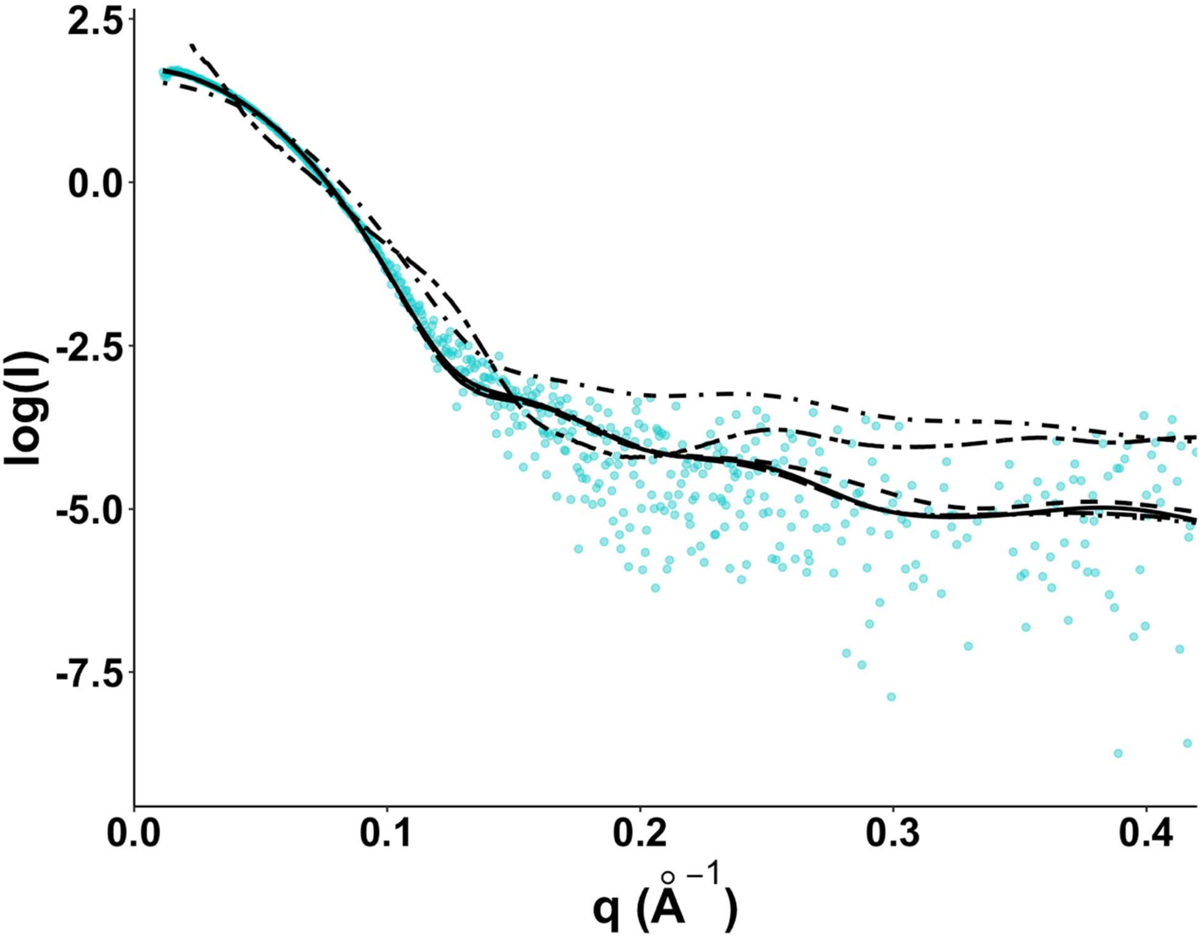

Figure S1 Fit of hCS SAXS data to crystal structures of citrate synthase. Predicted SAXS traces produced were fitted to the SAXS data in FOXS. PDB IDs used in fitting were 5UQU, 5UZP, 5UZQ, 6UZR, 6K5V, and 8GR8.

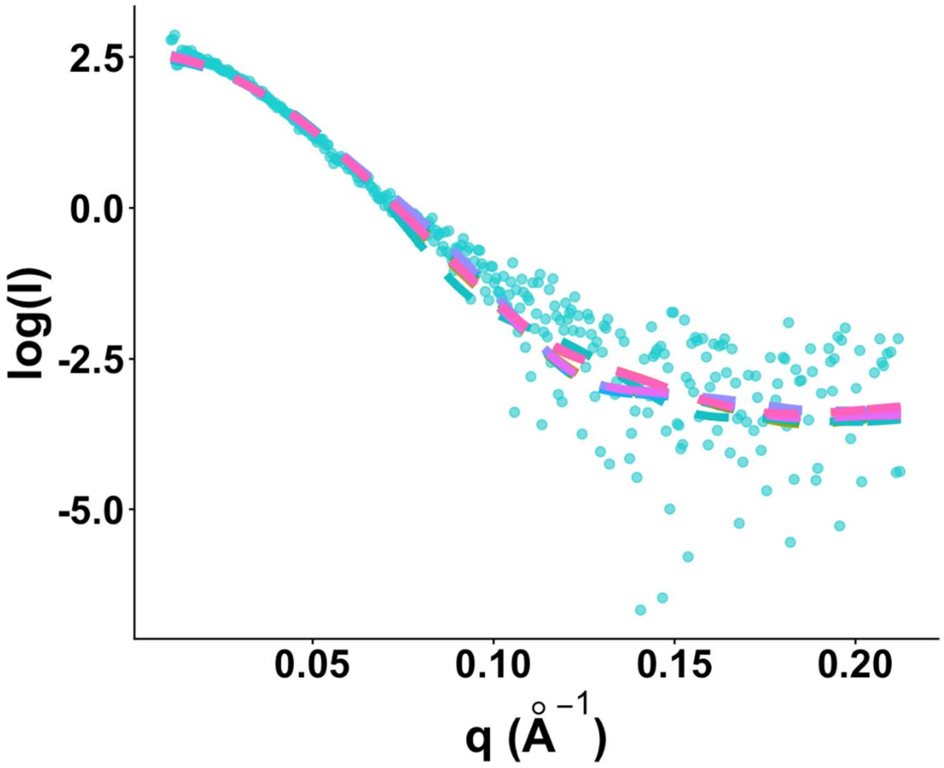

Figure S2 Fit of the Lightdock tetramers (lines) to the experimental SAXS data (cyan dots) from FOXS. Model fits had χ^2 values from 1.47 to 2.48.

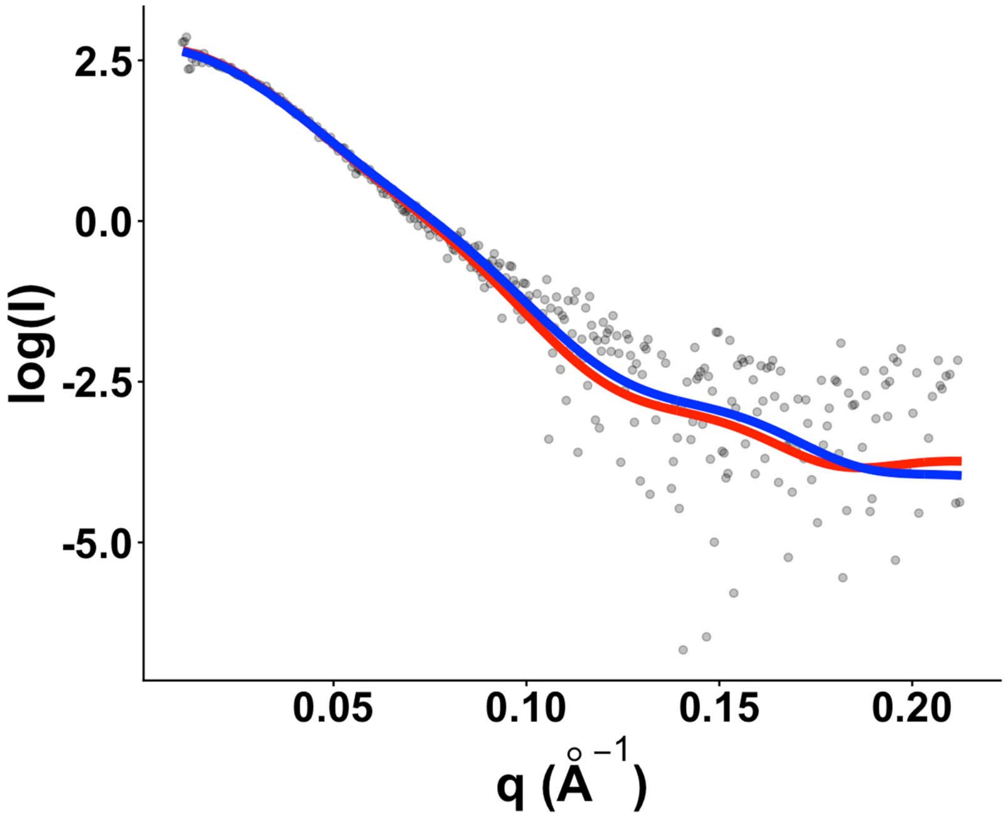

Figure S3 Multi-model fits from FOXS (red, χ^2 = 0.84) and OLIGOMER (blue, χ^2 = 0.93). The models used in the fit was the hMDH2-hCS hexamer, the best fitting hMDH2-hCS tetramer from Supporting Figure 2, and the individual dimers of hMDH2 and hCS. In the FOXS fit, the best fitting mix of species was an 85:15 ratio of hexamer to tetramer. In the OLIGOMER fit, the best fitting mix of species was an >99:1 ratio of hexamer to all other species.

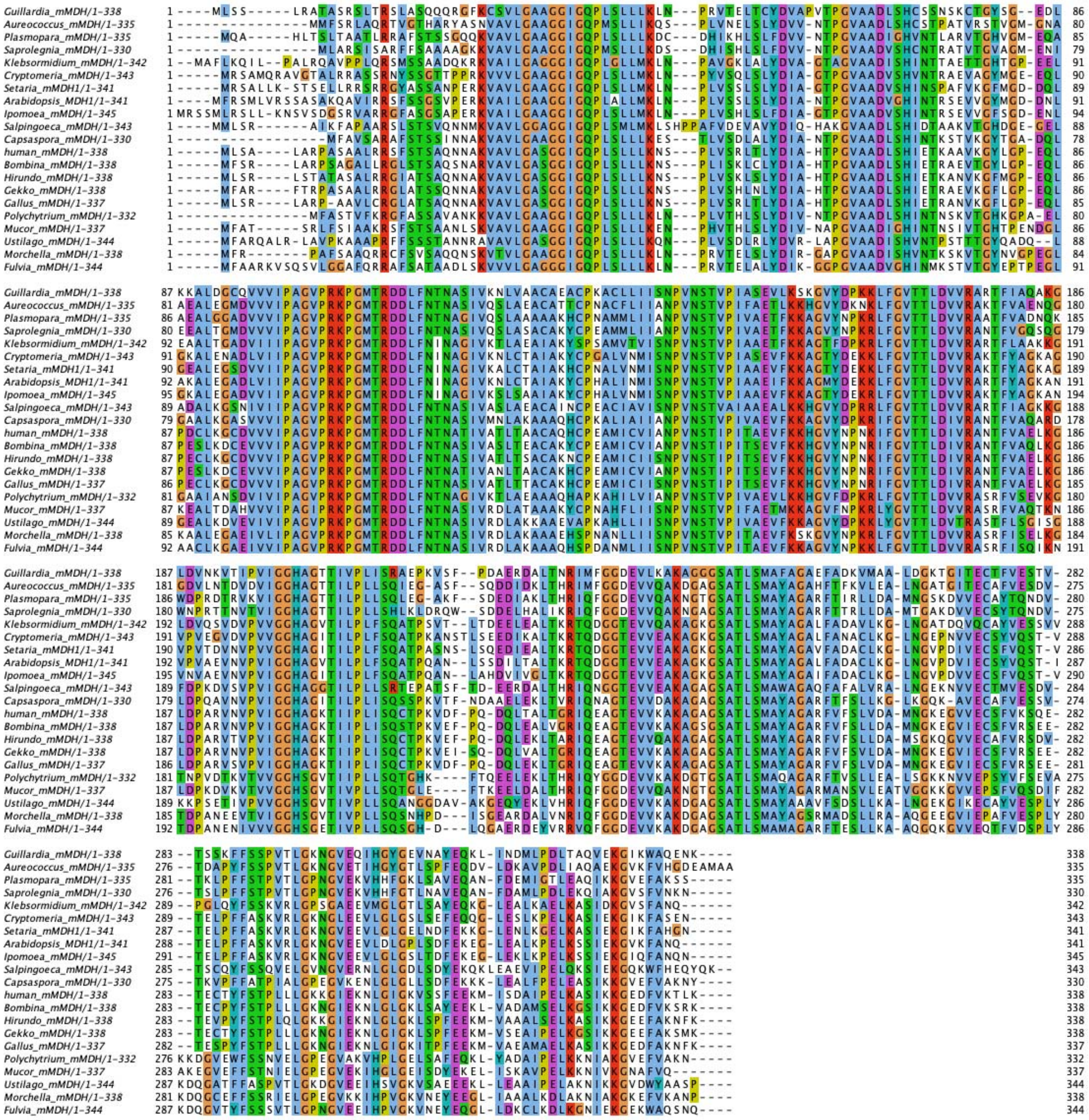

Figure S4 Alignment of Malate Dehydrogenase 2 homologs for ConSurf analysis.

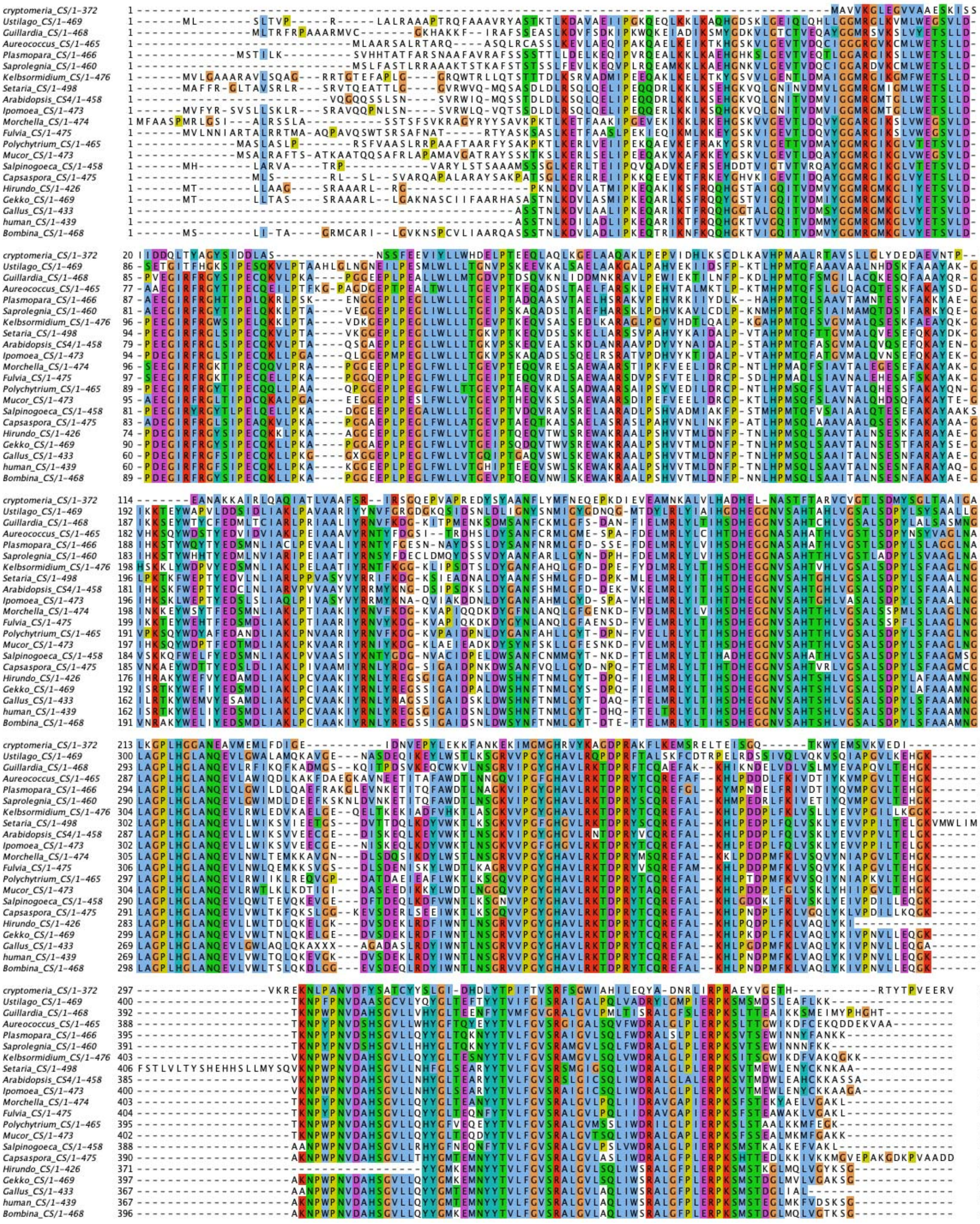

Figure S5 Alignment of Citrate Synthase homologs for ConSurf analysis.
